# Characterizing Styrene Monomer and Oligomers by SEC/MALS/VISC/DRI

**DOI:** 10.1007/s10337-023-04306-8

**Published:** 2024-02

**Authors:** André M. Striegel

**Affiliations:** 1 Chemical Sciences Division, National Institute of Standards and Technology (NIST), 100 Bureau Drive, MS 8390, Gaithersburg, MD 20899-8390, USA

**Keywords:** Styrene oligomers, Specific refractive index increment, Size-exclusion chromatography, Light scattering, Viscometry, Refractometry

## Abstract

Worldwide polystyrene (PS) production in 2020 was approximately 27 million metric tons, distributed among many nations, making it one of the most heavily imported and exported chemicals. Commercially produced PS usually possesses a broad molar mass distribution, often with a substantial oligomeric component. The latter can significantly affect processing and end-use, in addition to having potentially hazardous health effects and to impacting the polymer’s export classification by regulatory agencies. Quantitation of the oligomeric region of polymers by size-exclusion chromatography with concentration-sensitive and/or static light scattering detection is complicated by the non-constancy of the specific refractive index increment (∂n/∂c) in this region, which affects the calculated amount (mass fraction) of oligomer in a polymer, molar mass averages, and related conclusions regarding macromolecular properties. Here, a multi-detector SEC approach including differential refractometry, multi-angle static light scattering, and differential viscometry has been applied to determining the ∂n/∂c of n-butyl terminated styrene oligomers at each degree of polymerization from monomer to hexamer, and also of a hexadecamer. Large changes in this parameter from one degree of polymerization to the next are observed, including but not restricted to the fact that the (∂n/∂c) of the monomer is less than half that of PS polymer at identical experimental conditions. As part of this study, the individual effects of injection volume, flow rate, and temperature on chromatographic resolution were examined. Incorporation of the on-line viscometer allowed for accurate determination of the intrinsic viscosity and viscometric radius of the monomer and oligomers.

## Introduction

The worldwide production of polystyrene (PS) was estimated to have totaled 26.56 million metric tons in 2020 [1]. This figure is expected to increase by 5% to 10% annually over the next several decades. Global production of PS is distributed among many nations and, as such, it is both a heavily imported and heavily exported chemical. Commercially produced PS often possesses a broad molar mass distribution (MMD), covering several orders of magnitude in molar mass and extending from the oligomeric region up to millions of g mol^−1^. While macromolecular PS is relatively chemically inert, styrene monomer is a suspected cancer agent. One method by which to reduce these potential carcinogenic effects is to attach an alkyl group at one chain end (terminus) of the monomer.

Accurate characterization of the MMD and associated statistical averages is important for PS, as it is for virtually all polymers, to assess the accuracy of synthetic procedures as well as to optimize production processes and tailored end-use. Additionally, import and export of most macromolecules is contingent upon accurate quantitation of the oligomeric content of the material, generally regarded, for regulatory purposes [2], as the percentage of the MMD below 1000 g mol^−1^ and also below 500 g mol^−1^. This characterization is normally performed using size-exclusion chromatography (SEC) with on-line concentration-sensitive detection, most commonly differential refractometry (DRI). Absolute, *i.e.,* non-calibrant-relative, molar masses are obtained by adding either a static light scattering (SLS, most commonly multi-angle static light scattering or MALS) photometer and/or a viscometer (VISC) to the SEC detector train [3, 4]. A major obstacle to accurately determining the oligomeric content of a broad MMD polymer by one of the aforementioned SEC methods stems from the non-constancy of the specific refractive index increment (∂n/∂c) of polymers in the oligomeric region [5]. This parameter is fundamental to quantitating DRI and SLS response, as seen in Eqs. (1) and (2a), (2b), respectively:

(1) $$DRI_{\mathrm{resp}}=k_{DRI}\times c\times \frac{\partial n}{\partial c}$$

(2a) $$\frac{\Delta R(\theta )}{K^{*}c}=M_{w}P\left(\theta \right)\left[1-2A_{2}cM_{w}P\left(\theta \right)\right]$$

with

(2b) $$K^{*}=\frac{4\pi^{2}n_{0}^{2}(\partial n/\partial c{)}^{2}}{\lambda_{0}^{4}N_{A}}$$

In the above, $k_{DRI}$ represents the instrument constant for the particular refractometric piece of hardware employed, c is the concentration of analyte in solution, ΔR(θ) is the excess Rayleigh scattering factor (the amount of light scattered by the analyte solution in excess of that scattered by the solvent at angle $\theta ),\;M_{w}$ is the weight-average molar mass of the analyte, P(θ) is the particle scattering factor, $A_{2}$ is the second virial coefficient of the solution, $n_{0}$ the refractive index of the solvent at the experimental temperature and wavelength, $\lambda_{0}$ the vacuum wavelength of the incident radiation, and $N_{A}$ is Avogadro’s number. The refractometric measurement itself is a differential one (as is the static light scattering measurement), measuring the difference between the refractive index n of an analyte solution, in the limit of infinite dilution, vis-à-vis the refractive index $n_{0}$ of the solvent at identical experimental conditions. The ∂n/∂c is thus defined as:

(3) $$\frac{\partial n}{\partial c}\equiv \underset{c\to 0}{\lim}\frac{n-n_{0}}{c}$$

In a multi-detector SEC experiment involving VISC and DRI detectors, the ∂n/∂c also enters into the calculation of the intrinsic viscosity [η] of the analyte solution, defined as:

(4) $$\left[\eta \right]\equiv \underset{c\to 0}{\lim}\frac{\eta_{sp}}{c}$$

where the specific viscosity $\eta_{sp}$ is determined via the on-line VISC detector, and the concentration c by the DRI detector as per Eq. (1).

The reason for the molar mass (M) dependence of ∂n/∂c in the oligomeric region stems from the M-dependence of the refractive index of the polymer, which itself depends on density [6–8]. This density is affected by the concentration of chain ends in the polymer: at large M, said concentration is negligible compared to that of the main chain repeat units, so the chain ends have little (essentially no) effect on density and, consequently, on ∂n/∂c. At small M, not only is the concentration non-negligible but it changes sufficiently from one degree of polymerization to another so as to effect a change in ∂η/∂c between consecutive “mers,” *e.g*., between a dimer, trimer, and tetramer, or between a tetramer, pentamer, and hexamer. This non-constancy in ∂η/∂c in the oligomeric region is also a characteristic of absorptivity in this region, for the same reasons [9].

Given the large worldwide production and use of PS and its traffic across many borders, the latter especially but not exclusively necessitating accurate quantitation of the polymer’s oligomeric content, we have undertaken here the determination of the ∂n/∂c of individual akyl-terminated styrene oligomers, ranging from dimer to hexamer and including monomer and hexadecamer, at commonly employed experimental conditions. With these data in hand, it should be possible for researchers to more accurately determine the MMD and associated averages of broad distribution PS and to more accurately determine the oligomeric content of these samples. Determinations were performed using SEC/MALS/VISC/DRI and aided, in the case of monomeric styrene, by off-line, batch-mode DRI. Incorporation of the on-line viscometer allowed for highly accurate determination of the intrinsic viscosities of the analytes. The latter results were combined with the molar masses of the individual oligomers to yield a measure of analyte size, namely the viscometric radii $R_{\eta }$ of the oligomers.

## Experimental

### Materials

n-Butyl-terminated styrene monomer and oligomers were kindly provided by Agilent Technologies (Santa Clara, CA). Tetrahydrofuran (THF) stabilized with butylated hydroxyl-toluene (BHT) was from J. T. Baker.

### Oligomer Nomenclature

**Fig. 1:**
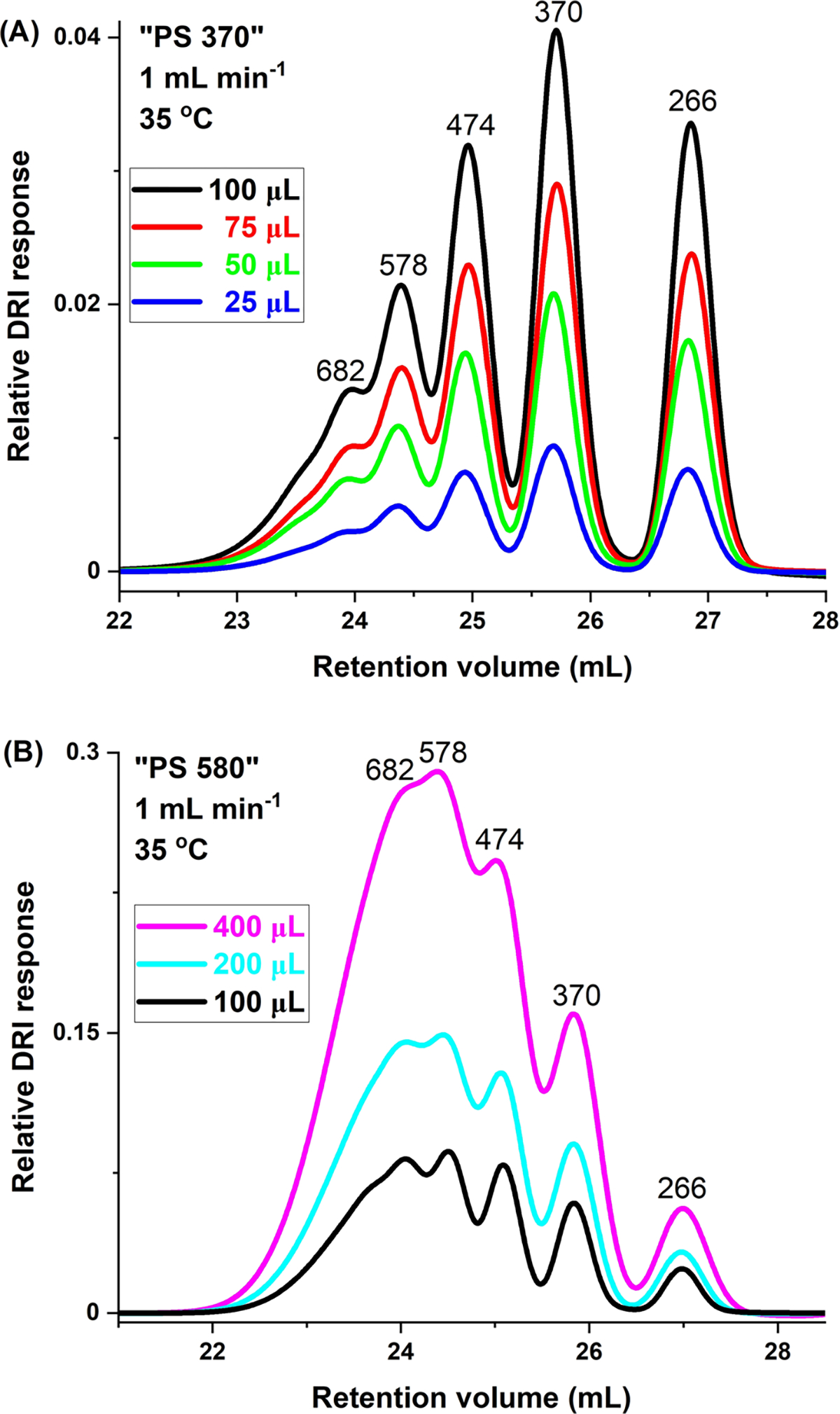
Effect of injection volume on $R_{s}$. SEC/DRI chromatograms of **A**) “PS 370” and **B** “PS 580.” Numbers above each peak indicate molar mass of each component oligomer, in g mol^−1^. Experimental conditions as given in figures. Relative DRI responses are comparable within, but not across, figures. VISC detector was part of the set-up in (**B**), but not in (**A**). All results based on at least triplicate determinations; in all cases, standard deviations are less than ± 1 in the last significant figure

**Scheme 1.**
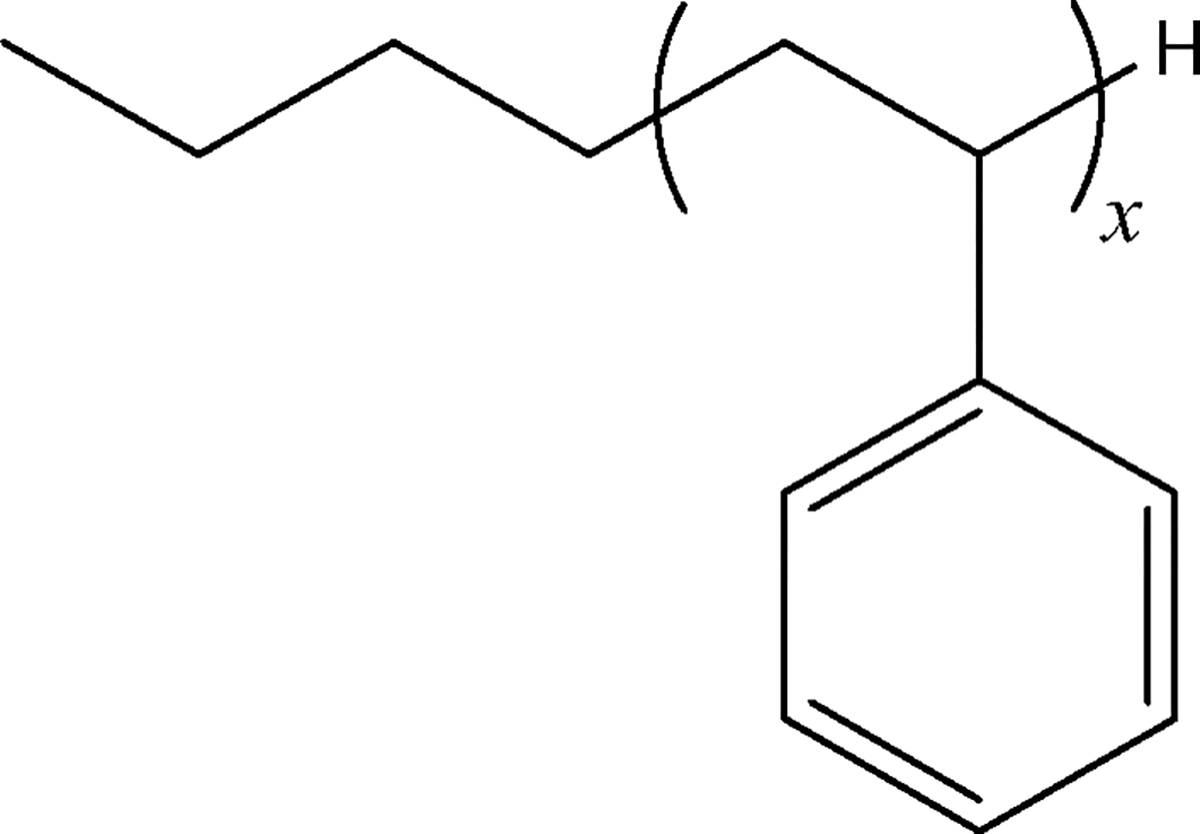
Structure n-butyl terminated styrene monomer and oligomers. For monomer, x=1

The particular monomer and oligomers analyzed here are all n--butyl terminated, as shown in Scheme 1. PS 162 is an n--butyl terminated styrene monomer (x=1). The samples referred to as “PS 370” and “PS 580” are each a collection of oligomers, in non-specified ratios; the quotation marks are meant to represent that these samples are not comprised a single mer, contrary to the case of PS 162. Quotation marks are also used because samples are sold under, and commonly referred to by, these names, which are representative of the most abundant oligomer in each mix (see Fig. 1). When quotation marks are not used, this means that results are for an individual mer. For example, “PS 370” refers to the sample containing several different styrene degrees of polymerization (mers), whereas PS 370 (without quotation marks) is the n--butyl terminated styrene trimer (x=3). “PS 1722” is referred to here as an n--butyl terminated styrene hexadecamer (x=16), though it is likely some oligomers with both higher and lower x are present in this sample.

### SEC/MALS/VISC/DRI Analysis

The SEC instrument consisted of an Agilent 1260 isocratic HPLC pump and autosampler (Agilent Technologies), the column oven of a Waters 2795 separations module (Waters Corp., Milford, MA), an on-line degasser connected to a set of four PLgel 5-μm particle size, 50-Å pore size styrene/divinylbenzene SEC columns, followed by a DAWN HELEOS-II MALS detector (Wyatt Technology Corp., Santa Barbara, CA), an optional ViscoStar III differential viscometer (Wyatt), and an Optilab T-rEX DRI (Wyatt). The viscometer was only employed when determining [ η ] and $R_{\eta }$; because it reduces by a factor of 2 the amount of sample going into the DRI, the viscometer was removed for on-line ∂n/∂c determinations by Method 3 (see Results and Discussion—Determination of Specific Refractive Index Increment (∂n/∂c)). A 0.2 μm Teflon filter (Whatman) was placed after the pump and prior to the injector; no sample filtration was performed prior to injection. In all cases the injector, column, and detector temperatures matched each other, with the exception of experiments at 50 °C for which the injector temperature was 40 °C. The MALS detector measures light scattered at 16 different angles simultaneously. Results presented here were usually derived from an angular measurement range spanning from 64° to 141°. The vacuum wavelength of operation $\lambda_{0}$ of the MALS detector is (658 ± 4) nm; that of the DRI matches this value to within 7 nm. Solution concentration of all analytes was 2 mg mL^−1^, except for “PS 1722,” for which it was 10 mg mL^−1^. The higher concentration of “PS 1722” versus those of the other analytes stems from the fact that “PS 1722” was employed for normalization of the MALS photodiodes as well as for determination of interdetector delays and interdetector band broadening. Data collection was performed using Astra software (Wyatt, version 7.3.2.21). Data were processed employing a first order Zimm formalism in Astra.

### Off-Line, Batch-Mode DRI of PS 162

As explained in the Results and Discussion, the ∂n/∂c of PS 162 was obtained by three different approaches. Given that, unlike “PS 370” and “PS 580,” PS 162 was a pure compound and not a blend of oligomers, its ∂n/∂c could be obtained by an off-line, batch-mode DRI approach (one of the three approaches). Because this is a sample-intensive procedure, it was not applied to “PS 1722,” for which little sample remained after using it for MALS normalization at the various experimental conditions explored. For off-line, batch-mode DRI, seven dissolutions of PS 162, ranging from 1 mg mL^−1^ to 8.7 mg mL^−1^, were injected directly into the same DRI detector as used in the SEC experiments using a Pump 11 Pico Plus Elite syringe pump (Harvard Apparatus, Holliston, MA) at a flow rate of 0.2 mL min^−1^. Detector temperature was 35 °C. Solutions were filtered through 0.20 μm syringe filters to remove potential contaminants in the solvent used; given the small sizes of the samples analyzed, no sample degradation is expected due to filtering. Data acquisition and processing were performed using Astra software (Wyatt).

### Determining Chromatographic Resolution $R_{s}$

For “PS 370” and “PS 580,” the peaks were fitted by Gaussian curves using the Peak Analyzer and Integrate functions in Origin 2022b (Northampton, MA). The method is described in more detail in reference [10]. Chromatographic resolution $R_{s}$ was calculated using Eq. (5):

(5) $$R_{s}=\frac{2\left(x_{c,B}-x_{c,A}\right)}{w_{A}+w_{B}}$$

where the subscripts A and B represent the first (earlier eluting) and second (later eluting) peak, respectively, in a peak pair; $x_{c}$ denotes the center of gravity of each peak; and w is the peak width at half height.

## Results and Discussion

### Effect of Injection Volume, Flow Rate, and Temperature on Resolution

SEC analysis of PS is quite often conducted using THF as solvent and mobile phase, at slightly above room temperature. Given the ubiquity of these solvent/temperature conditions, they were chosen here so that any information obtained could be of use to a broad cross-section of the scientific community working in this area. Prior to attempting to determine the specific refractive index increment, intrinsic viscosity, and viscometric radius of the styrene oligomers, we examined the effects of several experimental parameters, namely injection volume, flow rate, and temperature, on chromatographic resolution $R_{s}$ for “PS 370” and/or “PS 580.”

The effect of injection volume on $R_{s}$ is shown in Fig. 1A, B for “PS 370” and “PS 580,” respectively. The former overlays the DRI chromatograms at injection volumes ranging from 25 μL to 100 μL in 25 μL increments. The latter overlays the chromatograms for 100 μL, 200 μL, and 400 μL injection volumes. From Table 1 and Fig. 1A, we see that $R_{s}$ is essentially injection-volume-independent for the oligomers over the 25 μL to 100 μL range probed, with somewhat higher $R_{s}$ values at 100 μL, most notably for the tetramer-pentamer pair of PS 474–578. At larger injection volumes $R_{s}$ declines at the experimental conditions for all oligomer pairs, as noted by the decreasing $R_{s}$ values as a function of increasing injection volume in Table 2 and Fig. 1B. Based on these two sets of data, we opted for an injection volume of 100 μL in the present experiments.

**Fig. 2:**
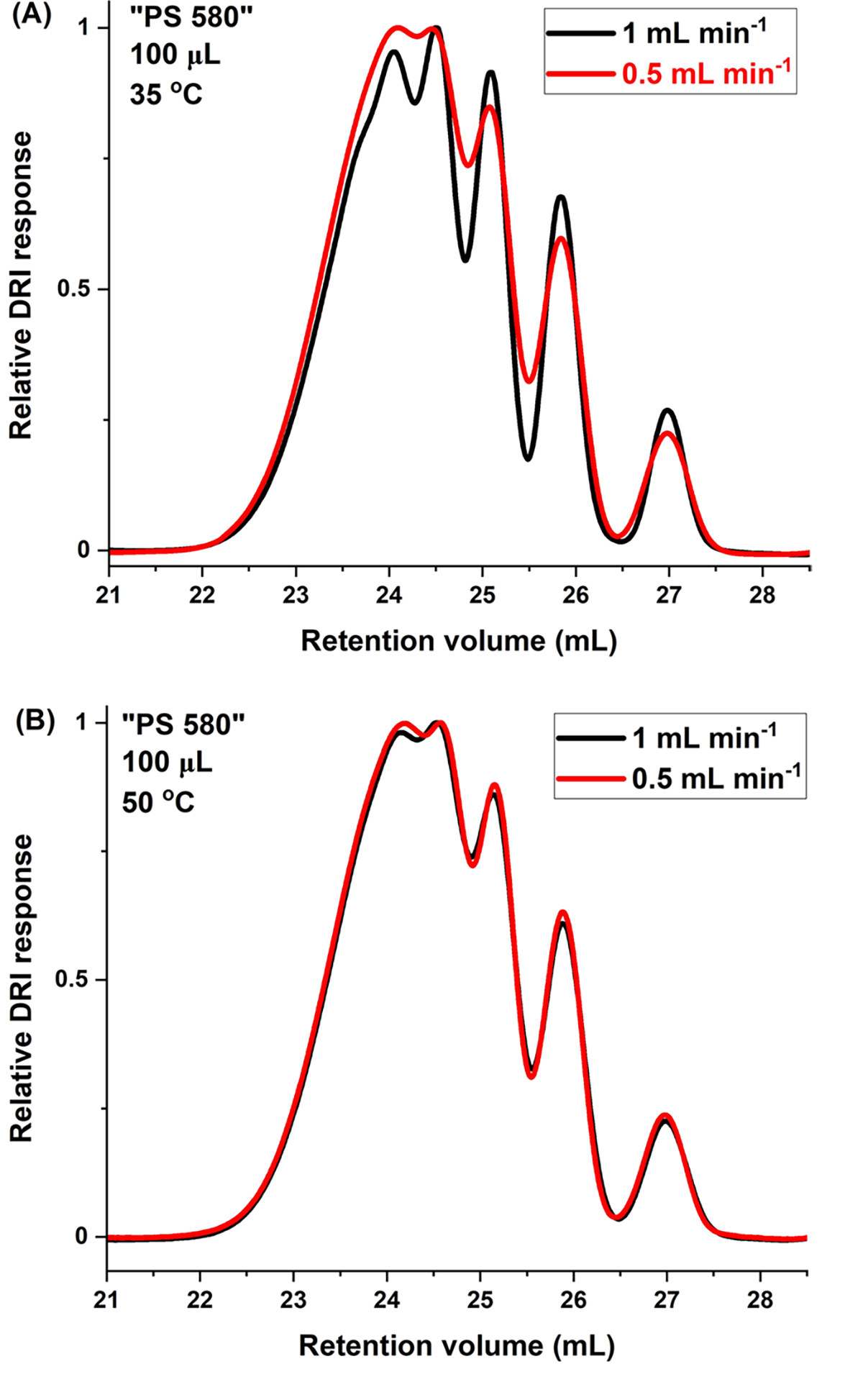
Effect of flow rate on $R_{s}$. SEC/DRI of “PS 580” at **A** 35 °C, **B** 50 °C. Experimental conditions as given in figures. In both cases, VISC detector was part of set-up

**Fig. 3:**
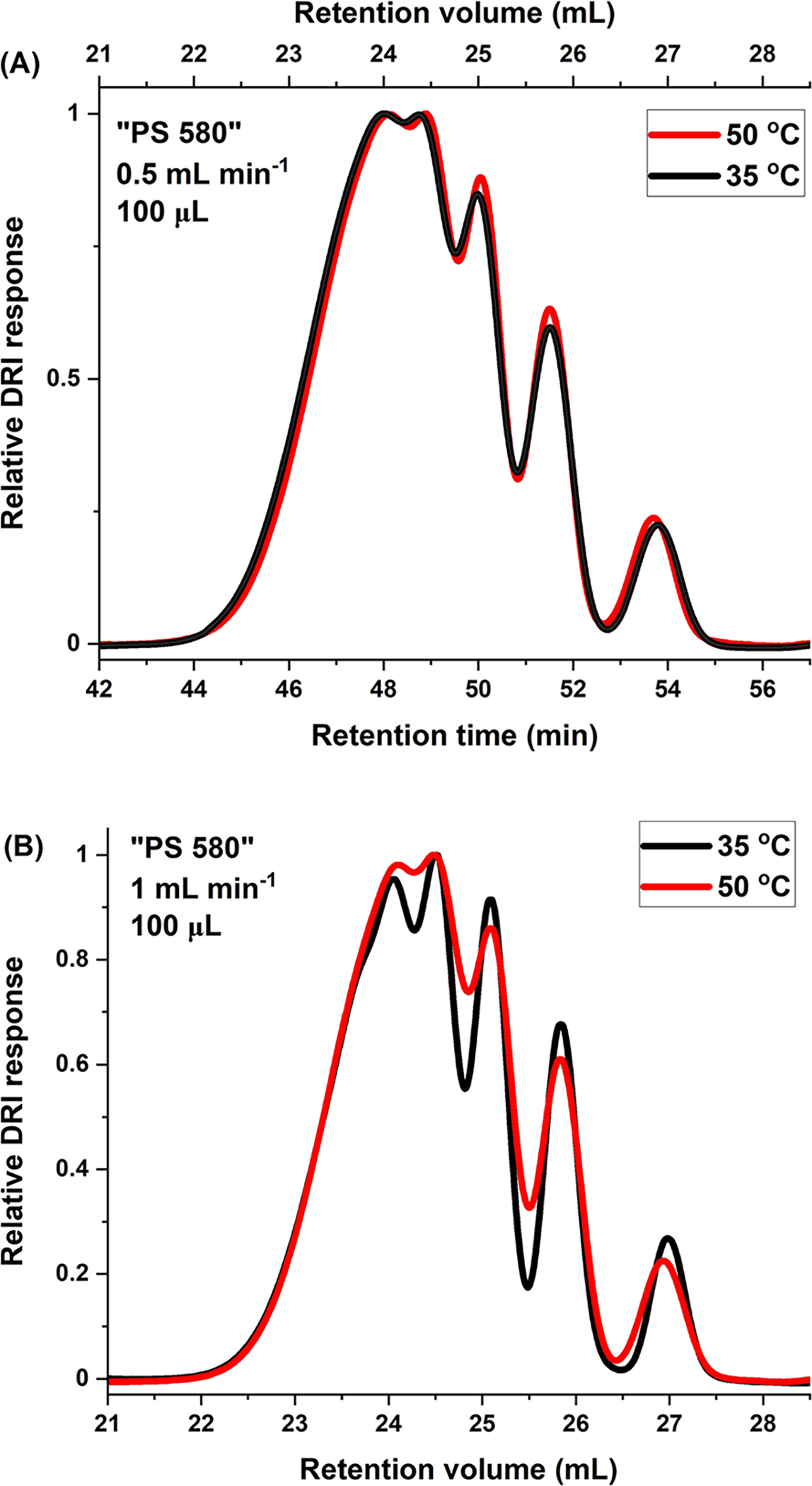
Effect of temperature on $R_{s}$. SEC/DRI of “PS 580” at **A** 0.5 mL min^−1^, **B** 1 mL min^−1^. Experimental conditions as given in figures. In both cases, VISC detector was part of set-up

The equilibrium nature of the SEC process is recognized by, inter alia, temperature- and flow-rate-independent retention [3, 11]. Both of these, especially flow-rate-independence, are observed best when plotting elution as a function of retention volume rather than retention time. For “PS 580,” the fact that retention does not depend on flow rate is seen, at both 35 °C and 50 °C, in Fig. 2A, B. It should be noted that, unlike retention, band broadening in SEC is affected by temperature. Figure 3A, B show the effects of temperature, 35 °C versus 50 °C, on elution for “PS 580.” As can be seen, retention is temperature-independent in all cases (the minuscule retention volume shift for the PS 266 dimer as a function of temperature in Fig. 3 is of ≈ 0.05 mL).

The effect of flow rate on $R_{s}$ in seen by comparing to each other either the two 35 °C or the two 50 °C columns in Table 3. At each temperature, $R_{s}$ are given at 1 mL min^−1^ and at 0.5 mL min^−1^. For the three oligomer pairs examined, $R_{s}$ is higher at 1 mL min^−1^ when measurements were performed at 35 °C. The inverse was found to be the case at 50 °C. Likewise, $R_{s}$ improved with decreasing temperature at 1 mL min ^−1^, but $R_{s}$ was better at the higher temperature at 0.5 mL min^−1^. Comparing all results in Table 3, $R_{s}$ was highest for all the oligomer pairs examined at 1 mL min^−1^ and 35 °C, so these were the flow rate and temperature conditions chosen for subsequent analyses.

### Determination of Specific Refractive Index Increment (∂n/∂c).

#### PS 162

PS 162 is an n-butyl-terminated styrene monomer the molar mass of which is 162 g mol^−1^ (x=1 in Scheme 1). Its ∂n/∂c was determined by three different methods:

##### Method 1:

Off-line, batch-mode DRI. The experimental details of this approach are given in the Experimental – Off-Line, Batch-Mode DRI of PS 162 section.

##### Method 2:

Assume 100% mass recovery. This method uses the on-line DRI chromatogram, with appropriately placed baseline and integration limits, and assumes that 100% of the injected sample eluted from the column and is contained within the integrated chromatographic peak. The instrument calibration constant is needed, as is the concentration of the injected solution and the injection volume. This method was employed recently to determine the ∂n/∂c of various methyl-α-pyranosides [12] and also of cello- and xylo-oligosaccharides and to demonstrate how, for the latter two series, the ∂n/∂c changes from one mer to the next within the oligomeric region [13].

##### Method 3:

Vary ∂n/∂c to match M. This approach is only valid for monodisperse oligomeric analytes for which the exact M is known.

Of the above approaches, Method 1 is generally considered the most accurate. As such, we employ it here, using PS 162 as a test analyte, as a benchmark to which we can compare results from the other two methods.

**Fig. 4:**
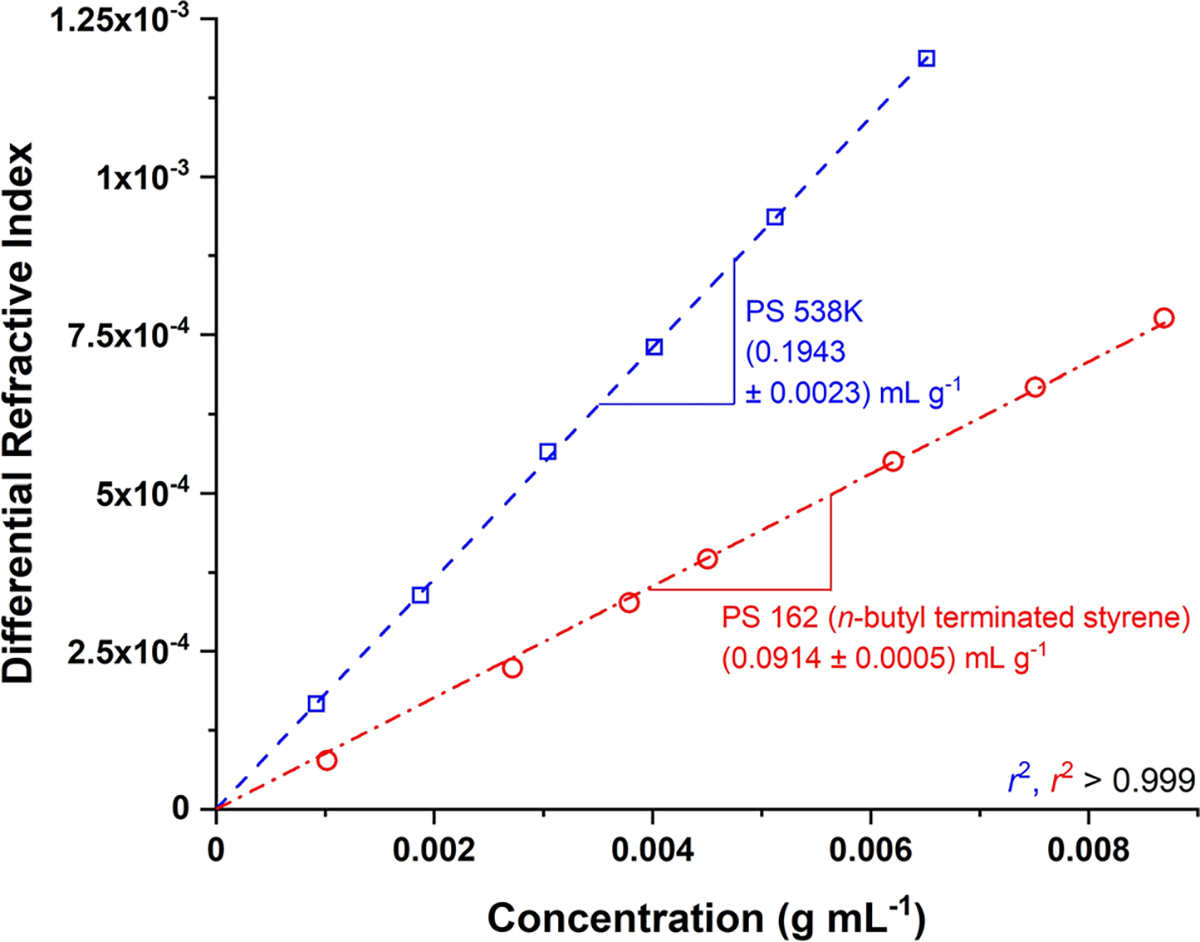
∂n/∂c plot of PS 162 (red) and of a typical high-M PS with $M=5.38\times 10^{5}\;g\;{\text{mol}}^{-1}$ (blue) at identical solvent, temperature, and wavelength conditions (THF, 35 °C, 658 nm, respectively). Symbols denote experimental results, straight lines are non-weighted first-order fits to the data. The slope of each fitted line corresponds to the ∂n/∂c of the respective sample at the experimental conditions. Instrumental standard deviations are smaller than data markers and, therefore, not shown

In Method 1, the differential refractive index of each injected solution, *i.e.,* the solvent-baseline-subtracted results for each of the sample dissolutions of different concentration, is plotted versus solution concentration. The slope of the plot is the ∂n/∂c of the sample at the particular solvent, temperature, and wavelength conditions of the experiment. At conditions identical to those employed here, the ∂n/∂c of many different high-M PS ($M\geq 7\times 10^{3}$ g mol^−1^) has been repeatedly measured to be (0.1943 ± 0.0040) mL g ^−1^ [14, 15]. An example of this is seen, for a narrow-dispersity PS with $M=5.38\times 10^{5}\;g\;{\text{mol}}^{-1}$, in blue in Fig. 4. Overlaid upon this, in red in the Figure, is the result for PS 162. As seen in Fig. 4, the ∂n/∂c of the latter was determined to be (0.0914 ± 0.0005) mL g^−1^. This is a value less than half that of polymeric PS. This means that, if one were to use the ∂n/∂c value for polymeric PS to calculate the molar mass of PS 162, one would underestimate the latter by more than a factor of two. Similarly, using the ∂n/∂c value for polymeric PS to try to ascertain the amount (mass fraction) of PS 162 in a sample would lead to underestimating this amount by more than a factor of two.

We next determined the ∂n/∂c of PS 162 using Method 2. Here, we used both SEC/MALS/DRI and SEC/MALS/VISC DRI and assumed that 100% of the injected analyte eluted from the columns and was contained within the integrated chromatographic DRI peak. Averaging over all sample injections, with and without the on-line viscometer, yielded a ∂n/∂c of (0.0896 ± 0.0004) mL g^−1^, within 2% of the result from Method 1.

Lastly, for Method 3 we adjusted the ∂n/∂c value for PS 162 in the Astra software, when processing the SEC/MALS/DRI data, to obtain a molar mass of 162 g mol^−1^ to within a variability of < 1 g mol^−1^. Using this approach, we obtained a ∂n/∂c of (0.0916 ± 0.0012) mL g ^−1^, also in excellent agreement with the value obtained from Method 1.

### PS Oligomers: Dimer Through Hexamer

The close agreement between the results for PS 162 from all three methods for determining ∂n/∂c is highly encouraging. The fact that the result from Method 3 agrees very closely with those from the other methods, especially from Method 1, provides confidence in the use of Method 3 for determining the ∂n/∂c of each of the individual oligomers comprising “PS 370” and “PS 580.” Each of these is a mix of PS dimer through hexamer, in unspecified ratios, with the most abundant oligomer in “PS 370” being its namesake trimer and the most abundant oligomer in “PS 580” being the pentamer with M = 578 g mol^−1^.

Employing Method 3 above along with the exact integer mass of each oligomer, the ∂n/∂c of each oligomer was determined from the SEC/MALS/DRI chromatograms of “PS 370” and “PS 580.” Center cuts of each chromatogram were employed which, combined with the above-determined $R_{s}$ values, translated to peak purities in excess of 98% in all cases [10]. The results for these analyses are given in Table 4. As can be seen from the table, the ∂n/∂c of the dimer is larger than that of the monomer PS 162, with an increase in ∂n/∂c continuing from one mer to the next. As compared to the ∂n/∂c of polymeric PS, the ∂n/∂c of the dimer is 35% lower. By the time the chain has grown to a hexamer, its ∂n/∂c is only 6% lower than that of the high-M polymer. It is important to note that each oligomer has its own ∂n/∂c, a value which would have to be incorporated into any molar mass or percentage amount calculations involving each individual oligomer if accuracy is desired.

The results presented here were obtained experimentally in THF at 35 °C employing DRI and MALS detectors for which $\lambda_{0}=658\;nm$. The ∂n/∂c values given in Table 4 are in very close agreement with the calculated ∂n/∂c of styrene monomer and of several styrene oligomers in THF at 25 °C for $\lambda_{0}=633\;nm$, as given in reference [16], and with the trend shown for styrene oligomers, though at unspecified solvent/temperature/wavelength conditions, in reference [17].

### PS Hexadecamer (“PS 1722”)

As mentioned earlier, “PS 1722” is referred to here as an n--butyl terminated styrene hexadecamer (x=16), though it is likely some oligomers with both higher and lower x are present in this sample. The ∂n/∂c of this analyte was determined employing both Methods 2 and 3, as described above. Results from both methods are statistically indistinguishable from one another. The average of all results is a ∂n/∂c of (0.1885 ± 0.0009) mL g^−1^ for “PS 1722,” only 3% less than that of polymeric PS at identical experimental conditions and very similar to the value reported for this sample in THF at 25 °C and $\lambda_{0}=633\;nm\;$ in reference [16].

**Fig. 5:**
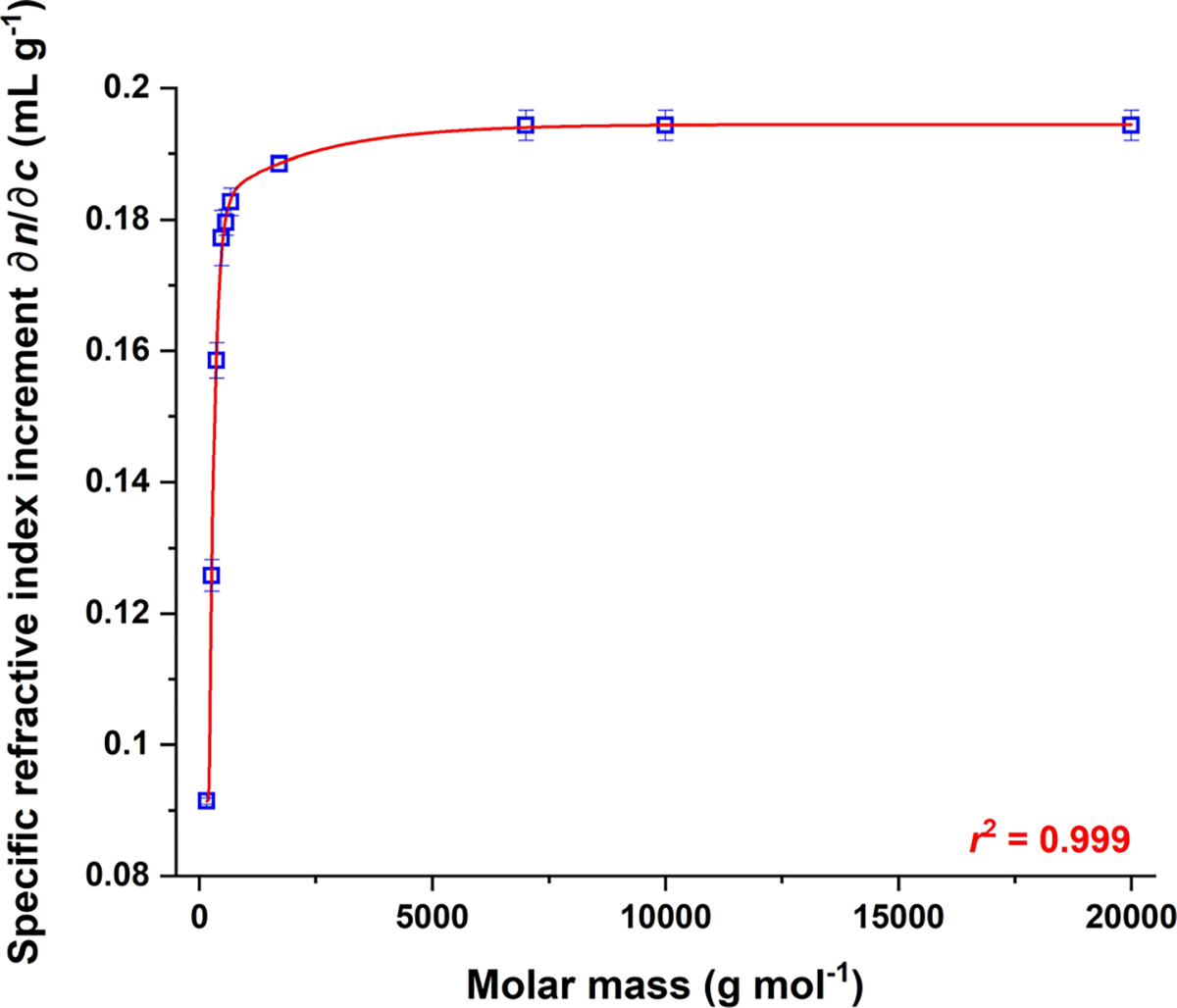
Change in ∂n/∂c with M for styrene monomer and oligomers, as compared to PS polymers. Determination of ∂n/∂c is described in text. Values for PS polymers are from the author’s laboratory, under identical experimental conditions (see, *e.g.,* reference [15]). Solvent: THF; temperature: 35 °C; $\lambda_{0}=658\;nm$. Solid red line corresponds to fit of results to Eq. (6)

The relationship between ∂n/∂c and molar mass for the styrene monomer and oligomers is shown graphically in Fig. 5, where the values for several polymer PS samples have also been included. The solid red line in the figure corresponds to a fit of the results to an exponential, specifically to a biphasic exponential association equation (“ExpAssoc2” fit in OriginPro 2022b):

(6) $$y=\left\{\begin{array}{c} Y_{b}+A_{1}\left[1-e^{-\left(\frac{x-TD_{1}}{\tau_{1}}\right)}\right],\text{for}\;xTD_{2}\\ Y_{b}+A_{1}\left[1-e^{-\left(\frac{x-TD_{1}}{\tau_{1}}\right)}\right]+A_{2}\left[1-e^{-\left(\frac{x-TD_{2}}{\tau_{2}}\right)}\right],\text{for}\;x\geq TD_{2} \end{array}\right.$$

where $Y_{b}$ is the baseline, *i.e*., the y-axis value at which the exponential begins; $A_{1}$ and $A_{2}$ are the first and second amplitudes, respectively, corresponding to the respective changes in response for the first and second exponentials; $TD_{1}$ and $TD_{2}$ are the first and second time offsets, respectively, corresponding to the respective x-axis values at which the first and second exponentials each begin; and $\tau_{1}$ and $\tau_{2}$ are the first and second time constants, respectively. The biphasic fit was employed to capture the ∂n/∂c of the n-butyl terminated styrene monomer PS 162 with the same curve as that of the styrene oligomers and PS polymers.

### Intrinsic Viscosity $\left[\eta \right.$] and Viscometric Radius $R_{\eta }$

**Fig. 6:**
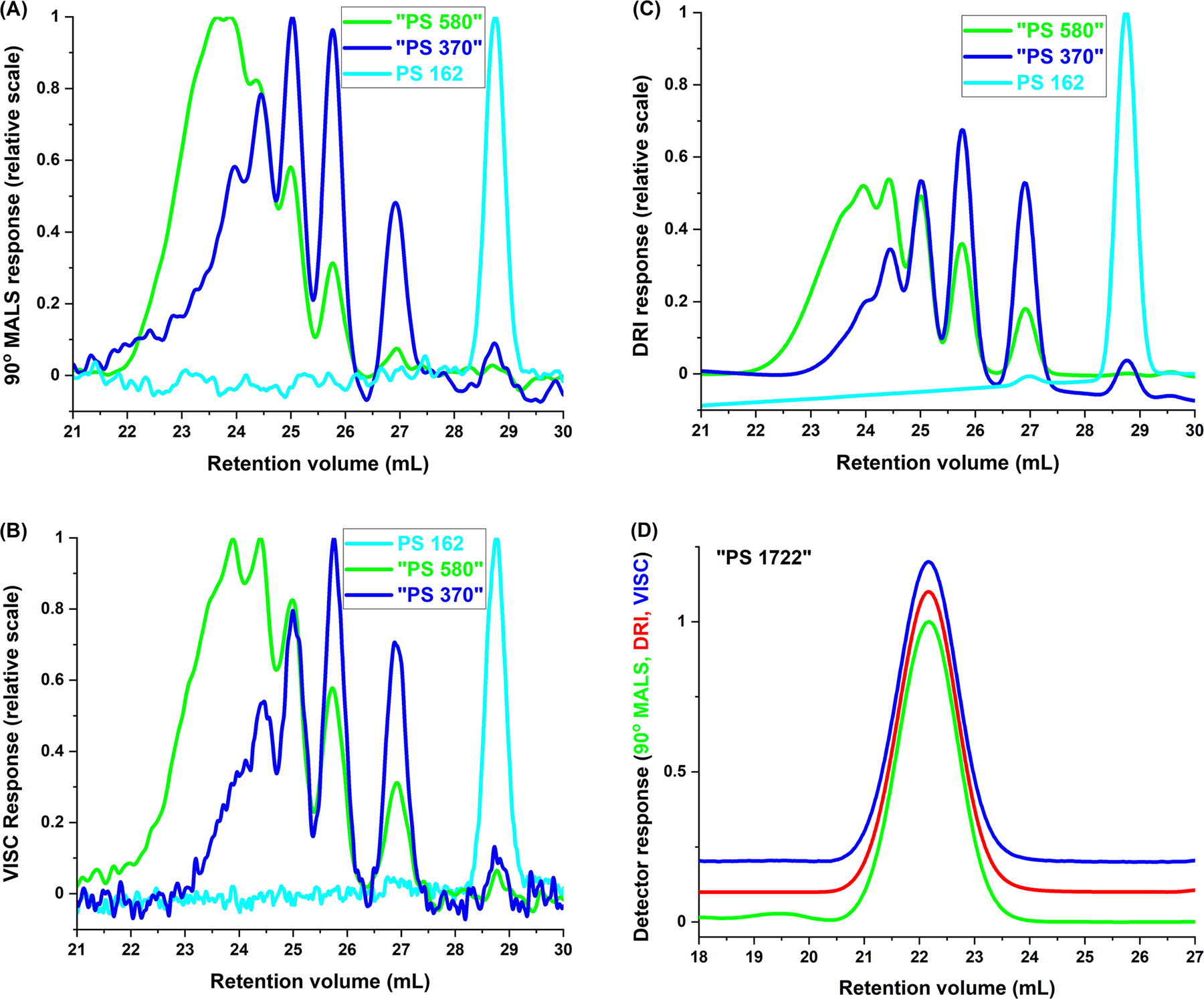
Overlay of **A** 90° MALS, **B** VISC, and **C** DRI chromatograms for “PS 580,” “PS 370,” and PS 162. **D** Overlay of 90° MALS, VISC, and DRI chromatograms for “PS 1722,” with y-axes offset from each other to ease visualization. Experimental conditions as described in text

Subsequent to the above experiments, an on-line viscometer was incorporated into the detector train, in the form of SEC/MALS/VISC/DRI. Overlays of the 90° MALS, VISC, and DRI traces for PS 162, “PS 370,” and “PS 580” are shown in Fig. 6A–C, while Fig. 6D is an overlay of these three detector traces for “PS 1722.” The latter analyte has been graphically separated from the former three because its solution concentration was substantially larger, and hence its detector signals are also substantially larger, than those of the other analytes owing to the fact that “PS 1722” was employed for normalization of the MALS photodiodes.

**Fig. 7:**
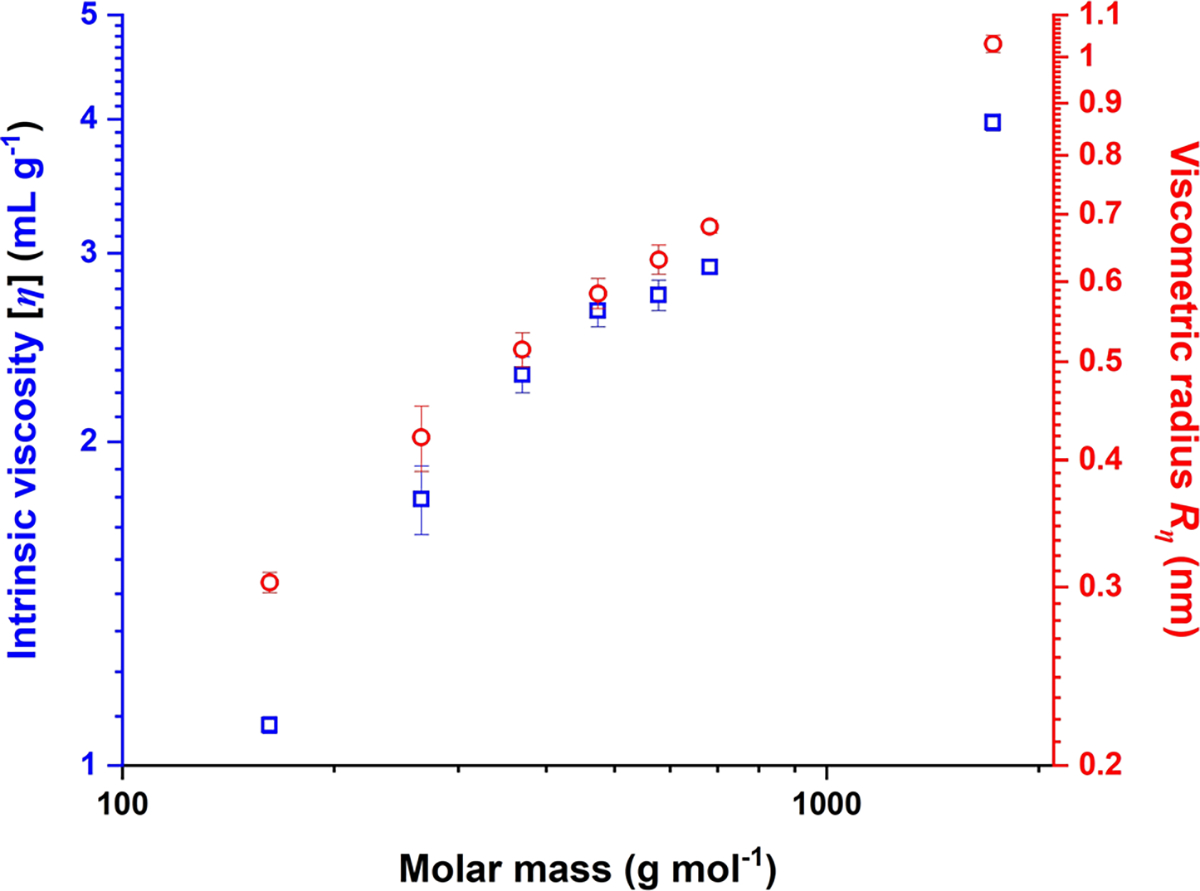
Mark-Houwink plot (blue) and $R_{n}$ conformation plot (red) of styrene monomer and oligomers. Experimental conditions as described in text. Standard deviations based on at least triplicate analysis of each sample

Incorporation of the viscometer allows determination of the intrinsic viscosity [η] of each analyte, as defined in Eq. (4). Results for this property for each styrene monomer and oligomer are given in Table 4. A so-called Mark-Houwink plot of molar mass versus intrinsic viscosity for the monomer and oligomers examined here, with each axis plotted on a logarithmic scale, is given in Fig. 7. As can be seen, the linearity associated with this type of plot for high-M linear PS at thermodynamically good solvent/temperature conditions has not been achieved yet at the oligomeric stage of chain growth. As noted in the Introduction, accurate determination of [η] depends on accurate knowledge of ∂n/∂c, because the latter is employed in calculation the solution concentration of the analyte, which is the denominator in Eq. (4). Errors in ∂n/∂c thus translate linearly into errors in [η].

A well-known macromolecular size metric is the so-called radius of gyration $R_{G}$, which is a measure of the root-mean-square distance of an atom or group of atoms in a molecule from the common center of mass [3, 4, 18, 19]. When MALS is used, $R_{G}$ is determined from angular dissymmetry experiments, *i.e.,* from measuring differences in the amount of light scattered by the analyte solution at one angle versus another. Monomers and oligomers, however, can be considered point scatterers: Within the measurement limits of modern MALS photometers these analytes scatter equally in all directions. This precludes measurement of $R_{G}$ for the styrene monomer and oligomers [3, 20, 21]. However, determination of [η] provides a means by which to gauge the size of these analytes by way of a different metric, namely the viscometric radius $R_{n}$, defined mathematically as [19]:

$$R_{\eta }\equiv {\left(\frac{3[\eta ]M}{10\pi N_{A}}\right)}^{1/3}$$

Conceptually, one may think of the $R_{\eta }$ as the radius of a homogeneous hard sphere that changes the viscosity of a solution by the same amount as does the analyte. Viscometric radii for the styrene monomer and oligomers examined here are reported in the last column of Table 4. Their relationship versus molar mass is shown in Fig. 7, in the form of a conformation plot (*i.e.,* with each axis plotted on a logarithmic scale).

## Conclusions

A multi-detector SEC/MALS/VISC/DRI approach was applied here to the study of n--butyl terminated styrene monomer and oligomers. As part of this, the individual influence of injection volume, flow rate, and temperature on chromatographic resolution was investigated. All analytes were observed to elute by a near-ideal, equilibrium, essentially temperature-independent (i.e., entropically controlled) size-exclusion mechanism.

The SEC method employed allowed for determination of the specific refractive index increment ∂n/∂c of the monomer through hexamer in the series, as well as of the hexadecamer. Values for this parameter are observed to change from one degree of polymerization to the next, owing to the influence of the changing chain end density between different oligomers. The ∂n/∂c of styrene monomer was found to be less than half that of its high-M PS counterpart. If the ∂n/∂c of the latter were applied in the study of the former, the molar mass of styrene monomer would be underestimated by greater than a factor of two, as would be the amount of monomer present in a sample. Given the relation between oligomeric refractive index and density, an alternative approach to the determination of ∂n/∂c could potentially involve the determination of density for each oligomer [22].

Incorporation of an on-line viscometer into the experimental set-up, in conjunction with the determined ∂n/∂c of each oligomer, allowed for accurate determination of the intrinsic viscosity and viscometric radius of each oligomer studied. The latter serves to provide a measure of how analyte size changes as a function of molar mass or degree of polymerization in the oligomeric region, wherein lack of angular dissymmetry precludes measurement of $R_{G}$ using on-line MALS.

The results presented here should allow for a more accurate calculation of the MMD and associated statistical moments for broad-dispersity PS with significant oligomeric content (perhaps similar to what was done in reference [13] in the analysis of birch pulp), quantitation of this content as a mass fraction of the entire sample, and for more accurate insights into the properties of the oligomers themselves. The method presented can be extended to the study of monomers and oligomers of other natural and synthetic macromolecules beyond the styrenic ones examined here, for both regulatory [2, 23, 24] and scientific purposes. Recent work, for example, has shown how degree of polymerization influences the torsional profiles of thiophene and furan oligomers [25] as well as the antibacterial action of guanylated oligomers [26].

## Figures and Tables

**Table 1:** Effect of injection volume on $R_{s}$: “PS 370”

Peak pair	Injection volume (μL)			
	100	75	50	25

266–370	1.70	1.66	1.65	1.62
370–474	1.12	1.08	1.13	1.13
474–578	0.89	0.83	0.71	0.62

Without VISC detector. Numbers of peak pair components refer to molar masses of oligomers in g mol^−1^, as per Fig. 1A. Flow rate: 1 mL min^−1^; temperature: 35 °C. All results based on at least triplicate determinations; in all cases, standard deviations are less than ± 1 in the last significant figure

**Table 2:** Effect of injection volume on $R_{s}$: “PS 580”

Peak pair	Injection volume (μL)		
	400	200	100

266–370	0.74	1.28	1.56
370–474	0.62	0.89	1.13
474–578	0.51	0.78	1.09

With VISC detector. Numbers of peak pair components refer to molar masses of oligomers in g mol^−1^, as per Fig. 1B. Flow rate: 1 mL min^−1^; temperature: 35 °C. All results based on at least triplicate determinations; in all cases, standard deviations are less than ± 1 in the last significant figure

**Table 3:** Individual effects of flow rate and temperature on $R_{s}$: “PS 580”

Peak pair	1 mL min^−1^		0.5 mL min^−1^	
	35 °C	50 °C	35 °C	50 °C

266–370	1.56	1.23	1.26	1.33
370–474	1.13	0.92	0.93	0.98
474–578	1.09	0.83	0.83	0.93

With VISC detector. Numbers of peak pair components refer to molar masses of oligomers in g mol^−1^, as per Fig. 1B. Injection volume: 100 μL. All results based on at least triplicate determinations; in all cases, standard deviations are less than ± 1 in the last significant figure

**Table 4:** Specific refractive index increment, intrinsic viscosity, and viscometric radius of styrene monomer and oligomers

x	Molar mass (g mol^−1^)	∂n/∂c (mL g^−1^)	[η] (mL g^−1^)	$R_{\eta }$ (nm)

1	162	0.0914 ± 0.0005	1.09 ± 0.02	0.303 ± 0.007
2	266	0.1258 ± 0.0024	1.77 ± 0.13	0.421 ± 0.031
3	370	0.1585 ± 0.0027	2.31 ± 0.09	0.514 ± 0.020
4	474	0.1772 ± 0.0042	2.65 ± 0.09	0.584 ± 0.020
5	578	0.1796 ± 0.0020	2.74 ± 0.09	0.631 ± 0.021
6	682	0.1827 ± 0.0021	2.91 ± 0.04	0.680 ± 0.010
16^a^	1722	0.1885 ± 0.0009	3.97 ± 0.07	1.03 ± 0.02

x corresponds to degree of polymerization, as per Scheme 1. Solvent: THF; temperature: 35 °C; $\lambda_{0}=658\;\text{nm}$

a Likely contains some chains with x>16 and x<16

## References

[1] Statista Research, March 24, 2023 https://www.statista.com/statistics/1192886/thermoplastics-production-volume-by-type-globally/

[2] Epa US (1997) Polymer exemption guidance manual, EPA 744-B-97–001. US EPA, Washington

[3] Striegel AM, Yau WW, Kirkland JJ, Bly DD (2009) Modern size-exclusion liquid chromatography, 2nd edn. Wiley, Hoboken

[4] Striegel AM (2005) Anal Chem 77(5):104A–113A

[5] Striegel AM (2022) Chromatographia 85:307–313 10.1007/s10337-022-04143-1 PMC9813945 36620523

[6] Hiemenz PC, Lodge TP (2007) Polymer chemistry, 2nd edn. CRC Press, Boca Raton

[7] Huber K, Bantle S, Lutz P, Burchard W (1985) Macromolecules 18:1461–1467

[8] Einaga Y, Abe F, Yamakawa H (1993) Macromolecules 26:6243–6250

[9] Striegel AM (2013) Anal Bioanal Chem 405:8959–8967 23887277 10.1007/s00216-013-7198-1

[10] Striegel AM, Striegel DA (2022) Chromatographia 85:65–72

[11] Striegel AM (2023). In: Fanali S, Chankvetadze B, Haddad PR, Poole CF, Riekkola M-L (eds) Liquid chromatography: fundamentals and instrumentation, 3rd edn. Elsevier, Amsterdam, pp 509–537

[12] Striegel AM, Trainoff SP (2021) Chromatographia 84:37–45

[13] Pitkänen L, Sixta H (2020) Cellulose 27(9217):9225

[14] Striegel AM (2017) Chromatographia 80:989–996 28860670 10.1007/s10337-017-3294-2 PMC5572220

[15] Striegel AM (2014) J Chromatogr A 1359:147–145 25085820 10.1016/j.chroma.2014.07.033

[16] Chance RR, Baniukiewicz SP, Mintz D, Ver Strate G, Hadjichristidis N (1995) Int J Polym Anal Charact 1:3–34

[17] Podzimek S (2005). In: Striegel AM (ed) Multiple detection in size-exclusion chromatography, ACS symp ser 893. American Chemical Society, Washington, pp 94–112

[18] Zimm BH, Stockmayer WH (1949) J Chem Phys 17:1301–1314

[19] Smith MJ, Haidar IA, Striegel AM (2007) Analyst 132:455–460 17471392 10.1039/b618177e

[20] Cotts PM (1997) Polym Mater Sci Eng 77:44–45

[21] Gridnev AA, Cotts PM, Roe C, Barth H (2001) J Polym Sci A Polym Chem 39:1099–1105

[22] Trathnigg B, Feichtenhofer S, Kollroser M (1997) J Chromatogr A 786:75–84

[23] ECHA (2023) Guidance for monomers and polymers, version 3.0, European Chemicals Agency, Helsinki, Finland

[24] Beskers T (2023) LCGC Europe 36(s6):16–19

[25] Perkins MA, Cline LM, Tschumper GS (2021) J Phys Chem A 125:6228–6237 34240869 10.1021/acs.jpca.1c04714

[26] Wyers D, Jirapanjawat T, Quinn JF, Whittaker MR, Greening C, Junkers T (2023) Polym Chem 14:2126–2134

